# Experimental Investigation of the Impact of Loading Conditions on the Change in Thin NiTi Wire Resistance during Cyclic Stretching

**DOI:** 10.3390/ma17184577

**Published:** 2024-09-18

**Authors:** Jonasz Hartwich, Sławomir Duda, Sebastian Sławski, Marek Kciuk, Anna Woźniak, Grzegorz Gembalczyk

**Affiliations:** 1 Department of Theoretical and Applied Mechanics, Silesian University of Technology, Konarskiego 18A, 44-100 Gliwice, Polandgrzegorz.gembalczyk@polsl.pl (G.G.); 2 Department of Mechatronics, Silesian University of Technology, Akademicka 2A, 44-100 Gliwice, Poland; marek.kciuk@polsl.pl; 3 Materials Research Laboratory, Faculty of Mechanical Engineering, Silesian University of Technology, Akademicka 2A, 44-100 Gliwice, Poland

**Keywords:** shape memory alloy, resistance, stretching, NiTi, martensite reorientation

## Abstract

This paper presents the results of an experimental study designed to evaluate the effect of repeated stretching cycles on the electrical resistance change in a NiTi alloy wire. In particular, tests were carried out to determine the effect of the type of loading on resistance change in the investigated wires. Wires with a diameter of 100 μm were used in the research. The experiment was carried out on a dedicated test stand designed for this purpose. During the test, the samples were subjected to 40 identical tensile cycles. The electrical resistance, sample elongation, and tensile force during successive stretching cycles were measured. The conducted research demonstrated the impact of elongation and reorientation of the structure on the resistance change in NiTi alloy thin wires. The research included a comparison of the effect of two different types of loading on the electrical resistance change in the sample. During cyclic stretching of a NiTi alloy sample with constant displacement, a decrease in electrical resistance was observed after each successive stretching cycle. Alternatively, when stretching with a constant force, the value of electrical resistance increased. In both types of loads, the greatest change in resistance value was observed at the initial cycles.

## 1. Introduction

Shape memory alloys (SMAs) represent a significant category of smart materials. The internal structural (phase) changes in SMA that are triggered by environmental stimuli result in changes to the properties of the alloy [1,2]. Additionally, the phase change from the martensite phase to the austenite phase is a contributing factor to the shape memory effect (SME), which is the capacity of the material to regain its original shape. The factor inducing the phase transformation in SMAs depends on the type (chemical composition) of alloy. In the literature, two types of SMAs are described. The first is the thermal shape-memory alloy (TSMA), in which the transformation is induced by temperature [3]. The second is the magnetic shape-memory alloy (MSMA), in which the transformation occurs under the influence of an external magnetic field [4,5]. Among the SMA alloys, the nickel-titanium alloy (NiTi) is of greatest scientific interest and has the highest number of existing applications. NiTi alloys are classified as TSMA group materials, indicating that the external factor affecting the transformation is the change in temperature of the alloy [6]. The main crystal structures observed in NiTi alloys are austenite, twinned martensite and detwinned martensite. In some commercially produced alloys, a rhombohedral (R) phase may also be observed [7,8]. In NiTi alloys, a monoclinic B19′ martensitic structure is stable at low temperatures [9]. As the temperature increases, the B19′ martensitic structure transforms into an austenitic phase with a regular structure B2, which is stable at high temperatures [10]. The transformation temperature depends on the chemical composition of the alloy. The most common temperatures for NiTi alloys are those of the beginning (As) and end of austenitic transformation (Af) and, similarly, for the reverse transformation, those of the beginning (Ms) and end of martensitic transformation (Mf). The transformation temperature depends on the chemical composition of the alloy: for the same atomic composition, the value of the Ms temperature is about 60 °C. As the nickel content in the alloy increases above 50%, this temperature decreases rapidly [11]. The characteristic transformation temperatures are also influenced by various heat or thermo-mechanical treatments. The way in which the material is produced also has a significant effect on the occurrence of the R (rhombohedral) phase. Depending on how the alloy is produced, the R phase can be an intermediary between martensite (B2-R-B19′) and austenite (B19′-R-B2) transformations. The R-phase is typified by a narrow hysteresis loop, small deformation level, and immediate response. A very important issue limiting the use of the R-phase is its low thermal stability [12]. The deformation processes associated with the R phase have an impact on the overall thermomechanical behavior of the wires [13]. The processes associated with the R phase have been the least explored among the various transformation processes occurring in the NiTi alloys. This is due, in part, to the fact that the activity of these processes is very difficult to detect, largely as a result of the small degree of crystallographic strain involved and the peculiar nature of the features associated with the R phase. The thermomechanical behavior of the R-phase in NiTi wires was successfully investigated by Šittner et al. [14] using electrical resistance measurements. Pu et al. [15] investigated the effect of artificial thermal cyclic treatment combined with ageing treatment on the phase transformation behavior and tensile superelasticity of NiTi alloys produced by the EB-DED method. They observed, during the ageing process, the occurrence of a two-stage martensitic phase transformation involving a one-stage R-phase transformation; moreover, due to the machining processes used, they obtained one of the best superelasticity of NiTi alloys produced by additively methods. The applied stress also affects the electrical resistivity of NiTi during phase transformation [16]. Another important mechanism associated with SMAs is the reorientation of the martensitic structure. This phenomenon is evidenced by the emergence of stress plateau when SMAs are subjected to loading below the Ms temperature. The reorientation is a consequence of deformation, which results in the formation of the detwinned martensitic structure. Martensite that has been detwinned can be deformed up to approximately 8% without the formation of slip bands or the movement of dislocations. When heated, such deformed martensite is transformed into austenite, which removes the martensite-induced deformations, causing the SME. This phenomenon occurs when the alloy temperature is below Af. Above the Af temperature, loading of the SMAs leads to stress-induced martensitic transformation (SIMT), which generates a superelastic effect (SE), with a lower and upper plateau typically presented for NiTi alloys [17]. The mechanical and electrical properties of NiTi alloys are significantly dependent on the crystalline structure. Structural transformations also affect the electrical resistivity of NiTi [18]. This relationship can be used to identify the different phases of NiTi alloys. This is useful because of the simplicity of resistance measurements. The electrical resistance value of NiTi alloys is the result of a number of different factors, including mechanical and thermal factors. In research conducted by Wu et al. [19], the resistance of NiTi shape memory alloy wire was examined during thermo-mechanical loading. The results demonstrated that both thermal and stress factors influence the electrical resistance change. Antonucci et al. [20] demonstrated, during dynamic heating and cooling of NiTi, that electrical resistivity is an excellent indicator of the crystal structure. Furthermore, Novák et al. [21] investigating the dependence of resistivity on mechanical and thermal factors in NiTi alloys showing an R-phase, demonstrating that the resistivity of the martensitic phase increases as a result of tension.

Load monitoring sensor systems are commonly based on the use of electrical resistance measurements [22,23]. The effect of phase transitions on the electrical resistance of NiTi alloys makes it possible to use these alloys as such sensors. Resendes et al. [24] confirmed the possibility of the use of a NiTi alloy in the role of a strain gauge for the detection of hip prosthesis dislocation. In the range of 7 to 12, the developed device showed the highest sensitivity. Jain et al. [25] investigated the possibility of using changes in the resistance of the NiTi alloy for flow detection. Hunek et al. [26] developed pressure sensors based on the dependence of the resistance of the NiTi wire on its elongation.

The purpose of this paper was to determine the assessment of the type of loading during cyclic stretching. In particular, tests were carried out to determine the effect of the type of loading on the resistance change in the wire. Two different types of loading were investigated: cyclic stretching with a constant displacement value and cyclic loading with a constant force. Microscopic and X-ray diffraction research was also carried out to gain a better understanding of the material used. In order to perform the presented research, a proprietary test stand was made together with software allowing the automation of measurements. The observed relationships between the deformation and stresses of the NiTi alloy wire and their electrical resistance may be useful in various sensor systems. The investigated phenomena may be applicable in a number of different applications, including systems for monitoring the number of load cycles, strain gauges, displacement sensors, and so forth. Additionally, the comparison of two different types of loading allows better requirements for the manufacture of such sensory systems.

## 2. Materials and Methods

In this paper, tests were performed on samples made from a commercially available NiTi alloy with the temperature of the end of austenitic transformation (Af) according to the manufacturer being 70 °C. The present paper investigates thin NiTi wires with a length of 100 mm and a diameter of 100 μm. The material properties of NiTi alloys differ depending on the phase. For example, Young’s modulus for martensite is 28 MPa, whereas it is 75 MPa for austenite. The same is also true for the electrical properties of the alloy, with a resistivity of 80 μΩ·cm in the martensitic phase and 100 μΩ·cm in the austenitic phase [27].

For the purposes of the research, the material used was examined in consideration of the microstructure; the chemical element composition was also assessed, and phase analysis was carried out. This type of research is intended to enhance the understanding of the parameters of the material being investigated and determine its suitability for particular applications [28,29].

**Figure 1:**
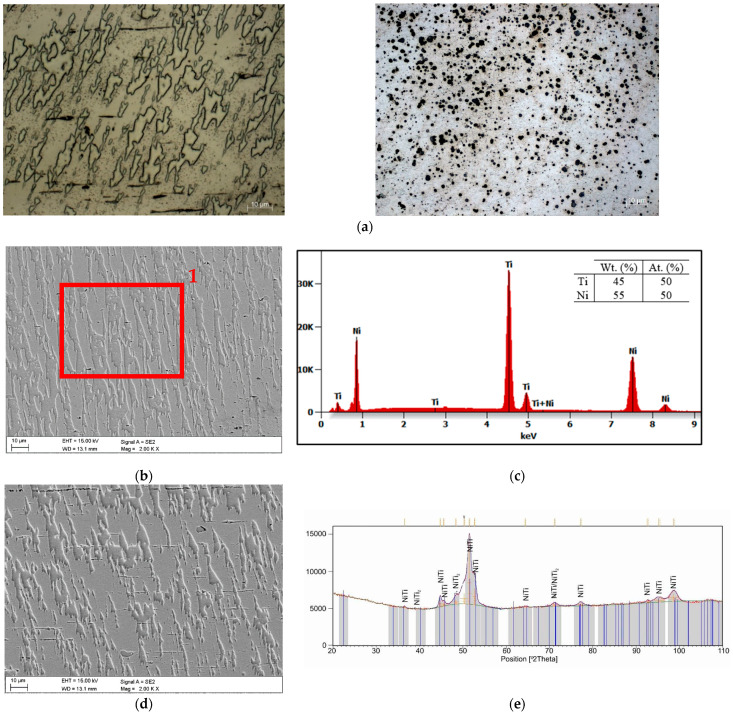
Results of microscopic observation: (**a**) OM, (**b**) SEM, (**c**) EDS—point 1 from Figure 1b, (**d**) SEM, (**e**) XRD diffraction pattern.

The microstructure and chemical composition were investigated using a Supra 35 (ZEISS AG, Oberkochen, Germany) high-resolution scanning electron microscope (SEM) and an energy dispersive X-ray detector (EDX, EDAX Trident XM4 AMETEK Inc. Berwyn, PA, USA). Phase analysis was completed using X-ray diffract, carried out using an ion X’Pert PRO X-ray diffractometer (Panalytical, Almelo, The Netherlands), using Co Kα (λ = 0.179 nm) radiation with 2θ ranging from 20 to 100° with a step of 0.05°. The obtained diffractograms were analyzed by means of X’Pert High Score Plus software (v. 3.0e) with a dedicated Inorganic Crystal Structure Database-ICSD (FIZ, Karlsruhe, Germany). Figure 1 presents the microstructure of tested NiTi-based SMAs determined by OM and SEM. It was found that the microstructures of the tested samples were dominated by dendrite grains. The microscopic observation (Figure 1a–d) and EDAX microanalyses showed that the matrix consists of a NiTi near-equiatomic phase. In addition, a certain fraction of NiTi_2_ particles and oxides can also be observed. The results of the XRD diffraction analysis confirm results of the microscopic analysis. As shown in Figure 1e, the XRD pattern indicates that tested samples consist of a dominant P21/m NiTi (B19′) phase with a monoclinic structure and Fd3m NiTi_2_ intermetallic compound with a face-cantered cubic structure. Similar results of NiTi alloy microstructure analysis are presented in the literature data [30,31,32,33].

Based on EDX, it was found that the detailed composition of the test material is as follows: nickel (Ni): 55 wt. %, 50 at.%; titanium (Ti): 45 wt. %, 50 at.%. The results of the material research indicate that the material is suitable for use in industrial applications. Moreover, tested material is often used in industrial applications.

NiTi alloy wires with a diameter of 100 μm were selected for the research investigated in this article. In the process of preparing the sample for the tests, the wires were cut into sections of equal lengths of 110 mm. Then, holders were attached to the ends of the wires to enable the wire to be attached to the test stand. At the same time, they provide an electrical contact for measuring the electrical resistance of the sample. The mounting of the holders consisted of three stages: first, the bent wire (in such a way that the operating length of the wire was 100 mm) was inserted into wire-end ferrules; in the next stage, the ferrules was clamped on the wire to prevent the sample from slipping out during testing; finally, the ferrules were connected to the eyelet connectors through a soldering process [34].

**Figure 2:**
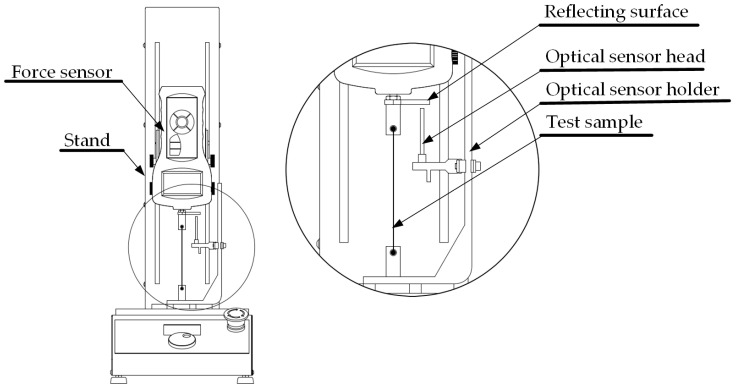
Scheme of the test stand with selected main components identified.

The properly prepared sample was mounted on the test stand. The first end of the specimen was mounted to the stand while the other end was mounted to the force sensor. The test stand with the attached sample is shown in Figure 2.

**Figure 3:**
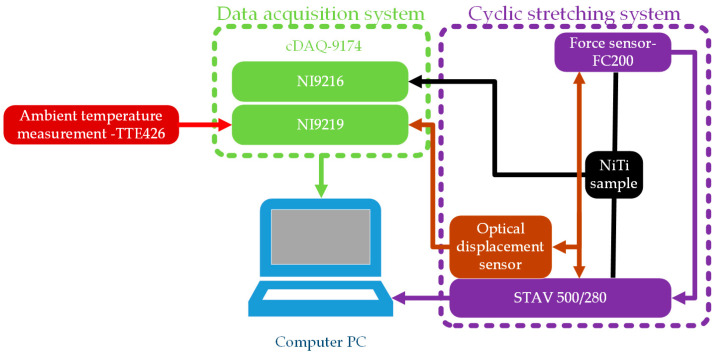
Schematic of the measurement system.

In the present paper, research was performed using a dedicated automated measurement system developed for this research. The most important elements of the test stand include the following: STAV 500/280 (AXIS Sp. z o.o., Gdansk, Poland), used for main movement (cyclic stretching) and sample mounting; an FC200 force sensor (AXIS Sp. z o.o., Gdansk, Poland), used for force measurements; an RC171 analog optical sensor (PHILTEC, Inc., Annapolis, MD, USA) (PHILTEC, Inc., Annapolis, MD, USA) mounted on a special holder, used for deformation measurements; and the TTE426 thermocouple, used for ambient temperature measurements. Data from the optical sensors and thermocouple were acquired using the NI9219 module (NI, Austin, TX, USA). Additionally, a NI9216 module (NI, Austin, TX, USA) was used for the 4-wire resistance measurements. For the collection of data from different measurement cards, the cDAQ-9174 (NI, Austin, TX, USA) data acquisition system was used. The measurement system was controlled via software developed in LabVIEW(2023 Q3). A measurement system schematic is presented in Figure 3.

The sample was cyclically stretched during the experiments, so the handle attached to the stand remained stationary, and the other end mounted on the force sensor moved with it by the movement made by the stand.

**Figure 4:**
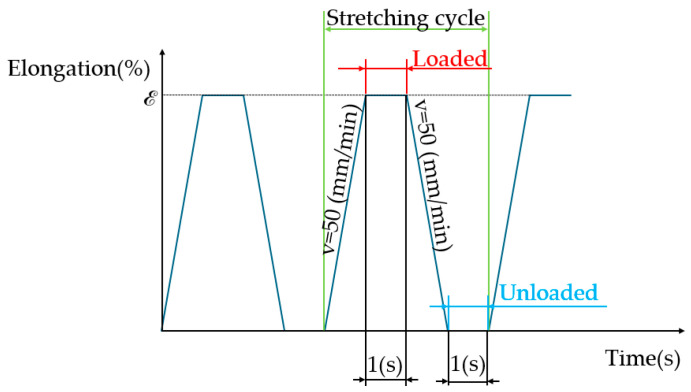
Schematic of the stretching cycle with constant deformation.

The tests were performed for two different types of loads. For the first load types, cyclic stretching was performed until a constant deformation value equal for each successive cycle was reached. For each sample, the assumed strain (ε) was 0.5% (0.5 mm). During each cycle, stretching was carried out at a constant speed (50 mm/min) until the system reached a remote deformation. It then remained in the achieved position for a time of 1 s before returning to the initial position at a constant speed (50 mm/min). After reaching the initial position, there was a pause lasting for 1 s. A schematic of the cycle with constant deformation is shown in Figure 4.

**Figure 5:**
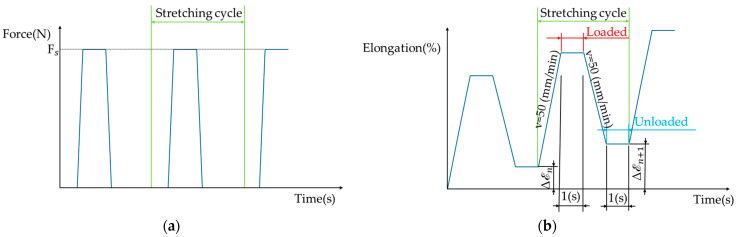
A schematic representation of the stretching cycle with a constant force: (**a**) set waveform of force; (**b**) expected waveform of displacement.

Additionally, tests were conducted to determine the efficacy of a second type of load. The constant value for this type of loading was the force applied to the sample. For each cycle, the force set (F_S_) value was 0.3 N. Therefore, the stand for the realization of the cycle required feedback not only from the drives but also from the force sensor. The force set value was similar to the forces achieved for the first cycle when stretching the samples with constant deformation. Analogous to the first load type, stretching was carried out at a constant speed (50 mm/min) until the force set value was registered by the force sensor. In the position, the system remained for a time of 1 s, after which it started the return at a constant speed (50 mm/min) until the force sensor registered a value of 0N. The system remained in this position for a period of 1 s. A schematic of the cycle with a constant force is shown in Figure 5.

In the constant force load type, after each successive cycle, the deformation relative to the base position (Δε) increases because the stand ends the cycle upon reaching the zero-force position. This is a consequence of the stress plateau observed in NiTi alloys.

The tests were carried out for 12 different samples (6 tested with a constant deformation load and 6 tested with a constant force load). The initial electrical resistance for each sample had a different value due to the following factors not being perfect: material fabrication, sample cutting and electrical contacts. Before starting the tests, each sample was submitted for transformation (martensitic–austenitic transformation was induced) in order to start measurements for the original shape. During the tests, each sample was subjected to 40 stretching cycles. The temperature during the tests oscillated between 25 and 26 °C.

## 3. Results

**Figure 6:**
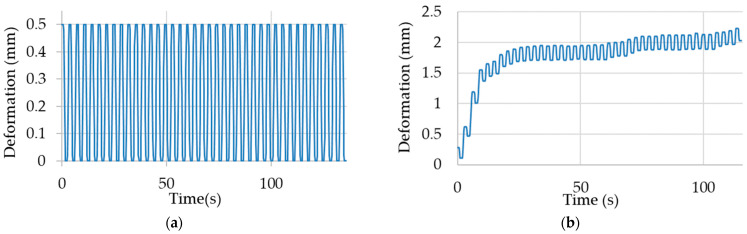
Example of deformation waveforms for the load type: (**a**) with constant displacement and (**b**) with constant force.

As part of the conducted research, the samples were subjected to cyclic stretching, during which the electrical resistance, deformation and force were measured. The deformation for the constant displacement load type in the unloaded stage of the cycles remained the same after each cycle, while for the constant force load type, it increased. This was due to a stress plateau, during which the strain could increase significantly with minimal stress change. A comparison of the sample deformation waveforms for the two different types of loads is presented in Figure 6.

**Figure 7:**
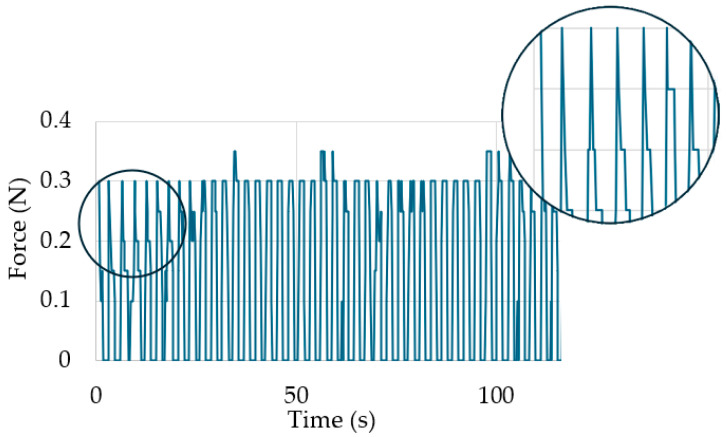
Example of the waveform of stretching force during cyclic loading with a constant force.

The increase in deformation relative to the base (Δε) for the first cycles was abrupt. However, it is notable that the change in deformation was considerably less with each cycle. The observed change in elongation may be attributed to reorientation of the sample’s internal structure, which involves a significant increase in strain with a minimal increase in stress. During cyclic stretching, fluctuations in force values and a decrease in resistance are observed during the loaded phase of the cycle. This decrease is greatest for the first cycles for those cycles, for which the largest change in the value of Δε is also observable. The waveform of the stretching force values during the measurements is presented in Figure 7.

**Figure 8:**
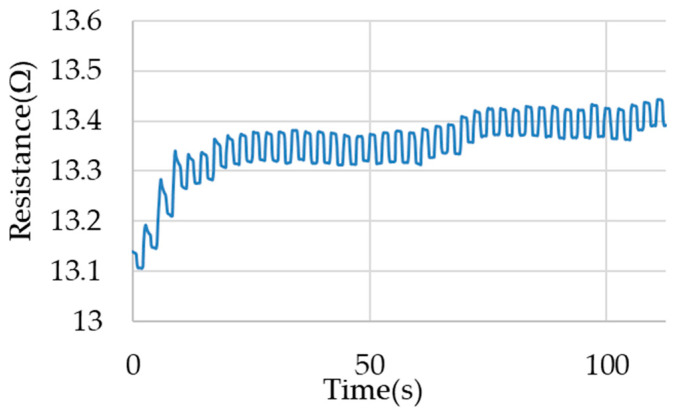
Example of electrical resistance waveforms for the load type with constant force.

For both types of loads, different responses of the electrical resistance of the sample for cyclic stretching are observed. For a wire loaded in the constant force load type, an increase in electrical resistance is observable after each successive cycle. This is an increase in value on the order of 0.05 Ω (for the first cycles) to 0.01 Ω (for later cycles). This increase occurs in a similar manner to the increase in deformation of the wire after successive cycles. The waveform of electrical resistance during the measurements is presented in Figure 8.

**Figure 9:**
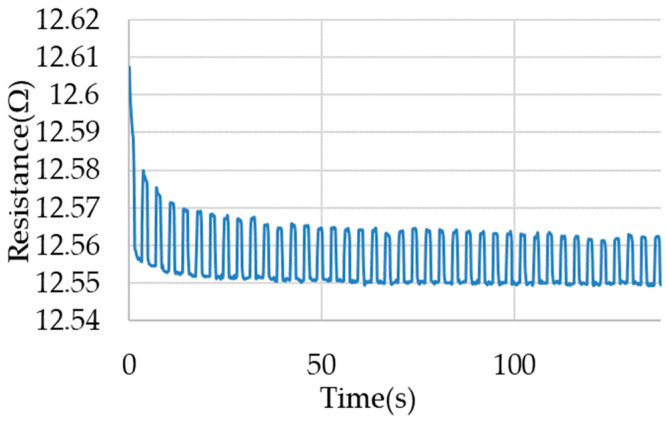
Example of electrical resistance waveforms for the load type with constant displacement.

For the second type of load with constant displacement, a different effect is observed. After each successive stretching cycle, the electrical resistance value of the sample decreases. This decrease is significant during the first stretching cycles, though it subsequently decelerates with each successive cycle. The value of the decrease in electrical resistance for this load type is also an order of magnitude smaller than the change in resistance for the constant force load type. The course of the electrical resistance value of the sample during stretching in the constant displacement load type is presented in Figure 9.

## 4. Discussion

**Figure 10:**
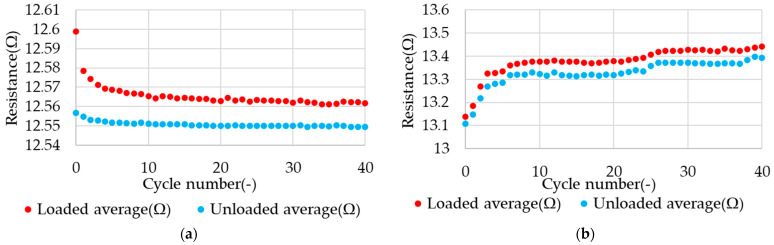
Example of average values of electrical resistance in relation to the stretching cycle for the load type (**a**) with constant displacement and (**b**) with constant force.

In order to perform a detailed analysis of the results, each cycle was divided into two phases (for both types of loads): loaded and unloaded. For both phases (duration of 1 s) of each cycle, the average value of the electrical resistance of the samples was determined. A comparison of the average electrical resistance values during the stretching cycles for both types of loads are presented in Figure 10.

**Figure 11:**
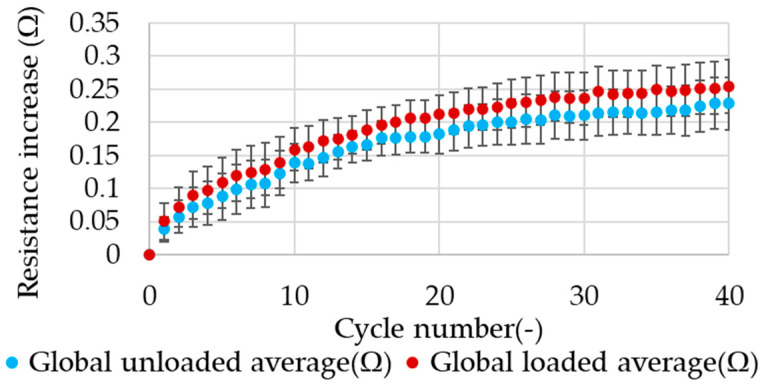
The average effect of cyclic stretching on electrical resistance with a constant force.

These trends were observed in all tested samples for the constant displacement load and for the constant force load. Due to the difference in the initial electrical resistance value between samples, the average resistance values in successive cycles were related to the resistance before the first cycle. This method permitted the comparison of the value of the change in resistance during cyclic stretching between samples for a particular load type. In the load type with a constant force for all samples, an increase in the average value of electrical resistance was observed for both phases of cycle. However, it was noted that there were variations in the values of the change in electrical resistance for different samples after each cycle. This is associated with the considerable standard deviation observed in the mean electrical resistance values across all samples, particularly in relation to the number of stretching cycles. The determined value of the standard deviation of the average resistance value exceeds the value of the difference between the loaded and unloaded stages of the cycle. The average value of resistance change for all tested samples in relation to the number of stretching cycles is presented in Figure 11.

**Figure 12:**
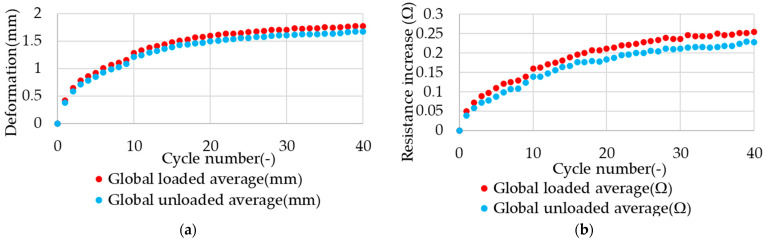
Averaged effect of cyclic stretching in constant force load type on the value of (**a**) deformation and (**b**) electrical resistance change in the samples.

In the case of the constant force load type, the observed trend is present in both the resistance value and the deformation of the test sample. A comparison of the averaged results demonstrates that analogous characteristics are observed for both of the measured values. The correlation between the electrical resistance of the NiTi alloy and its deformation has previously been observed in studies [35,36] and is likely to be observable for the present case. The correlation between the change in resistance for this type of loading and the deformation of the sample also results in a notable increase in the standard deviation. The impact of minor discrepancies on the length of the sample and the resulting discrepancy in the initial resistance value is more significant in terms of the final outcome. Furthermore, the discrepancy between the resistance values observed in the loaded and unloaded phases of the cycle is notably smaller than the increase in resistance values following subsequent stretching cycles. This results in a notable increase in the standard deviation of the electrical resistance values following each stretching cycle, which is larger than the difference between the cycle phases. This observable effect presents difficulty in utilizing it as a strain counter, as it requires the construction of a sensor system with high accuracy. However, it remains a viable option for strain measurement due to the significant correlation between resistance and strain. The occurrence of this observed trend for both of the measured values is more clearly discernible when the averaged values are compared after each successive cycle. A comparison of the aforementioned data is presented in Figure 12.

**Figure 13:**
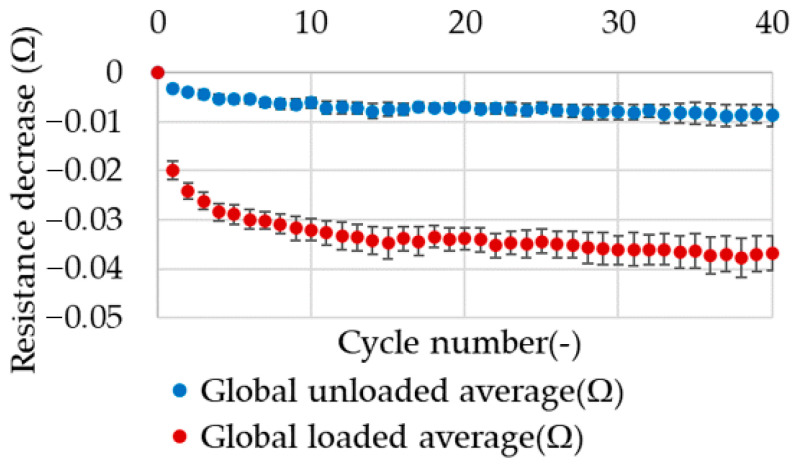
The average effect of cyclic stretching on electrical resistance with constant displacement.

A decrease in electrical resistance was observed following each successive stretching cycle during cyclic stretching with a constant displacement load type. This trend was observable for all samples. This can be demonstrated by the averaging of the decrease in electrical resistance values after each successive cycle. The results obtained in the load type with constant displacement demonstrate a markedly reduced value dispersion, as evidenced by the notably smaller standard deviation of the average value after each cycle. The minimum data dispersion is observed in the unloaded phase of the cycle for the averaged results. The average value of the resistance change for all tested samples in relation to the number of stretching cycles is presented in Figure 13.

The observed effect of a decrease in resistance during cyclic stretching can only be marginally related to the deformation of the sample. This is because both the elongation of the wire and the decrease in diameter (for thin NiTi alloy wires, this is an insignificantly small decrease) should be associated with an increase in resistance, as observed in the constant force load type. Therefore, the observed decrease in resistance cannot be related to the deformation of the sample. It seems most probable that the observed effect is related to the reorientation of the internal structure of the sample induced by stress.

In both types of loads, the effect of cyclic stretching is most significant in the initial cycles and subsequently diminishes with each successive cycle. In both cases, a reorientation of martensite structure is observed, whereas for a constant force, no decrease in resistance is evident; instead, there is an increase. This is probably due to the influence of other factors on the results, with the deformation of the sample having the most significant impact. The change in deformation of the sample after each cycle exerts a greater influence on the change in resistance than the reorientation of the internal structure. Consequently, the impact of the transformation is not discernible. Furthermore, the greater impact of deformation on the results may account for the larger dispersion of data observed in samples subjected to constant force stretching. It is possible that manufacturing inaccuracies may affect the deformation value after successive stretching cycles.

## 5. Conclusions

The conducted research demonstrated the impact of deformation and reorientation of structures on the resistance change in NiTi alloy thin wires. The research included a comparison of the effect of two different types of loading on the electrical resistance of the sample. A comparison was made between cyclic stretching of the wire with constant displacement (i.e., the sample in each cycle was stretched to reach a set position) and with a constant force (i.e., the sample in each cycle was stretched to reach a set force). During cyclic stretching of a NiTi alloy sample with constant displacement, a decrease in electrical resistance was observed after each successive stretching cycle. Alternatively, when stretching with a constant force, the value of electrical resistance increased. This is presumably due to the fact that different factors exert varying influences on the observed phenomena. When subjecting a sample to constant force load type, it can be observed that the extent of deformation increases with each successive cycle. This phenomenon is associated with an increase in the electrical resistance of the sample. In the case of stretching with constant displacement, the deformation of the sample is practically negligible (with the exception of the initial cycle), thus having a negligible effect on the resistance value in subsequent stretching cycles. Conversely, the reorientation of the martensite structure may represent a significant factor influencing the resistance of the sample in the case of constant displacement stretching. In both types of loads, the greatest change in resistance value is observed during the initial cycles. With each successive cycle, the observed changes are diminished, yet they remain quantifiable. The observed phenomena permit the degree of structural fatigue to be monitored, specifically in applications with low load dynamics.

## Data Availability

Data are contained within the article.
